# Carotid plaque score and ischemic stroke risk stratification through a combination of B-mode and contrast-enhanced ultrasound in patients with low and intermediate carotid stenosis

**DOI:** 10.3389/fcvm.2023.1209855

**Published:** 2023-12-18

**Authors:** Fang Li, Shi-Yao Gu, Lu-Ni Zhang, Jing Chen, Ming-Hua Yao, Ting-Ting Wu, Ji Ma, Cai-Xia Jia, Rong Wu

**Affiliations:** Department of Ultrasound, Shanghai General Hospital, Shanghai Jiao Tong University School of Medicine, Shanghai, China

**Keywords:** carotid plaque, ischemic stroke, risk stratification, contrast-enhanced ultrasound, B-mode ultrasound

## Abstract

**Objective:**

The occurrence of ischemic stroke (IS) is closely related to the characteristics of carotid plaque (CP). Due to the effect of stroke risk stratification based on B-mode ultrasound (US) and contrast-enhanced ultrasound (CEUS) that has not been studied in patients with low and intermediate carotid stenosis, we construct and validate a CP score and ischemic stroke risk stratification (ISRS) using a combination of B-mode and CEUS, in order to provide new convenient strategies to stratify these patients to prevent stroke.

**Materials and methods:**

This retrospective study evaluated 705 patients with low and intermediate carotid stenosis who underwent B-mode and CEUS from November 2021 to April 2023. Qualitative B-mode and CEUS features of carotid plaques were analyzed using a univariable and multivariable logistic regression to construct the CP score. Then, we combined the CP score with Essen stroke risk score (ESRS) to develop ISRS.

**Results:**

This study included a total of 705 patients with low and intermediate carotid stenosis, of which 394 were symptomatic patients (with a mean age of 71.03 ± 10.48 years) and 311 were asymptomatic patients (with a mean age of 65.13 ± 10.31 years). Plaque echogenicity, plaque morphology, carotid intima-media thickness in B-mode US and intraplaque neovascularization grading and perfusion pattern in CEUS were significantly associated with IS. The ISRS incorporating these five predictors and ESRS showed good discrimination and calibration in both primary cohort [area under the curve (AUC), 0.91; Hosmer–Lemeshow test, *p* = 0.903] and validation cohort (AUC, 0.84; Hosmer–Lemeshow test, *p* = 0.886).

**Conclusion:**

We developed an effective and practical tool to identify and stratify patients with low and intermediate carotid stenosis, based on the CP score and ISRS estimation. Our study may provide new insights into managing patients with no indication of surgery.

## 1. Introduction

Ischemic stroke (IS) is one of the leading causes of death and disability worldwide (Global Burden of Disease Study, 2019 Collaborators, 2019) (1). Atherosclerotic plaque rupture is a major cause of IS, accounting for 18%–25% of all strokes (2). A previous study reported that a high percentage of patients with low and intermediate stenosis experienced ischemic events during follow-up (3). An extensive research showed that the risk of IS in patients with carotid stenosis is independently correlated with plaque vulnerability (4). Accurate screening tools should be developed to identify patients at different levels of cerebrovascular risk, especially those with low and intermediate vascular stenosis.

Carotid intima-media thickness (C-IMT) is one of the recognized indicators of atherosclerosis and is linked to an increased risk of stroke (5). The hypoechoic and irregular plaque in B-mode ultrasound (US) often shows histological features of plaque instability, such as the presence of lipids and higher expression of inflammatory mediators (6). Contrast-enhanced ultrasound (CEUS) enables the objective visualization of intraplaque neovascularization (IPN) by clearly indicating active inflammation, which is an inherent feature of vulnerable plaque (7). The characteristics of US imaging make it an ideal imaging method for screening patients with carotid atherosclerosis to find vulnerable plaques (8).

MRI is the imaging modality of choice to identify the majority of vulnerable plaque components (9); however, it has drawbacks such as a long procedure time and many contraindications (10). Computed tomography (CT) has shown a high sensitivity and specificity for the detection of ulcerated plaques and intraplaque hemorrhage (11). But the disadvantages of CT for plaque assessment are the radiation exposure and need for iodinated contrast (12). A quick and easy method to categorize plaque that can anticipate the development of carotid lesions and upcoming ischemia episodes is required. In this study, we established the carotid plaque (CP) score according to the parameters of B-mode US and CEUS. The Essen stroke risk score (ESRS) is a simple and easy tool used to operate a stroke risk stratification scale in clinical practice (13). We combined the CP score with ESRS to develop an effective and practical tool to identify patients at risk of cerebrovascular events and to stratify them, in order to provide new strategies to prevent IS in patients with low and intermediate carotid stenosis.

## 2. Material and methods

### 2.1. Study population

This is a retrospective study, which consecutively reviewed patients diagnosed with carotid atherosclerosis between November 2021 and April 2023. The patients were divided into primary and validation cohorts in a chronological order. The patients between October to November 2021 and December 2022 made up the primary cohort, and those between January 2023 and April 2023 were in the validation cohort. All patients with low and intermediate carotid artery stenosis who received magnetic resonance angiography (MRA) or computed tomography angiography (CTA) examinations and clinical follow-up using B-mode US or CEUS were included in this study. The study was approved by the medical ethics committee of Shanghai General Hospital, Shanghai Jiao Tong University School of Medicine [No. (2022) 028].

**Figure 1:**
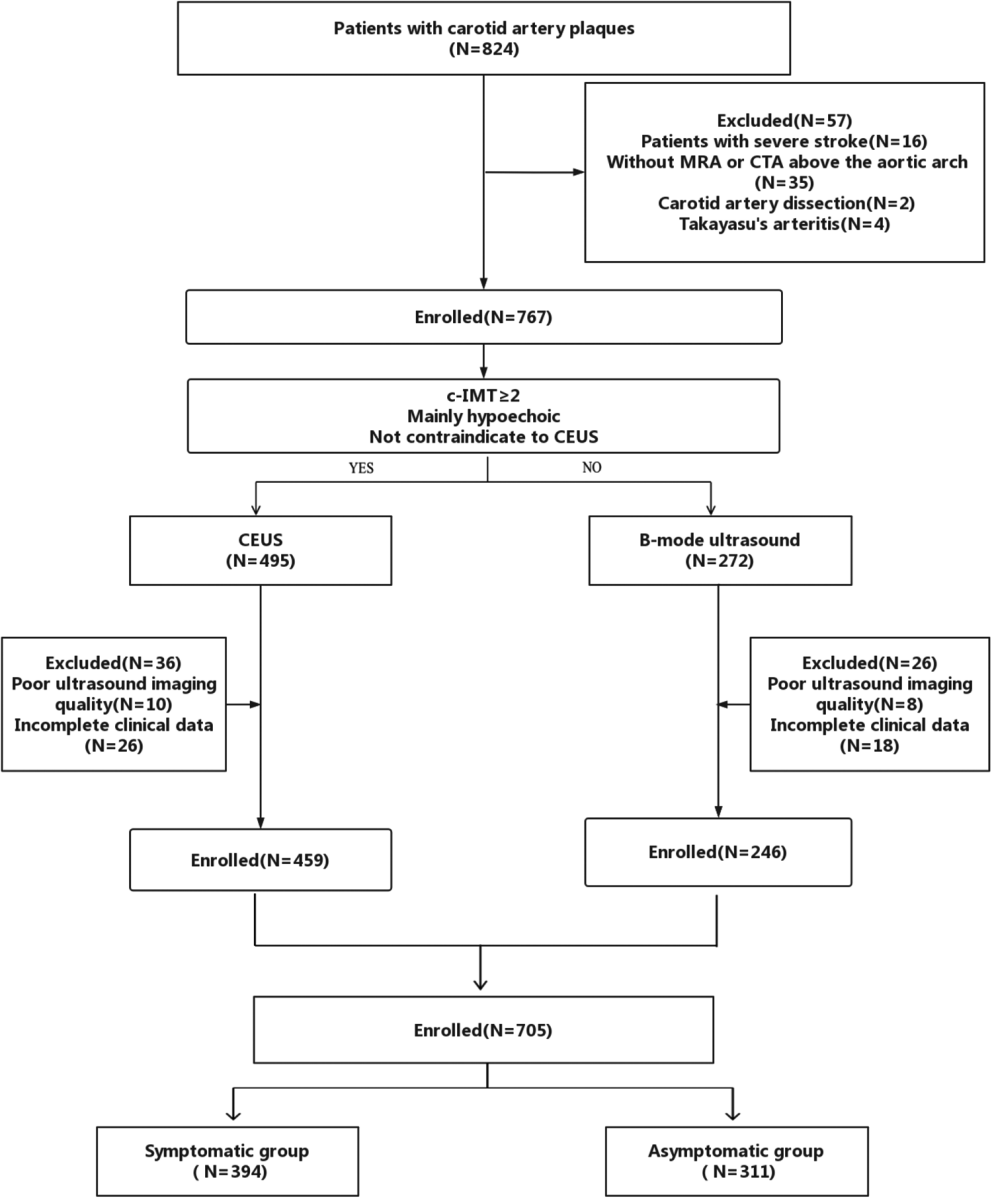
Flowchart of patient enrollment.

The inclusion criteria included (1) ≥18 years of age, (2) low and intermediate carotid stenosis, and (3) no clinical contraindications for CEUS. The exclusion criteria included (1) severe stroke, (2) without MRA or CTA above the aortic arch, (3) carotid artery dissection, (4) Takayasu's arteritis, and (5) poor image quality of B-mode US and CEUS. We selected plaques with a thickness of ≥2 mm and that were mainly hypoechoic, as hyperechoic plaques with calcification cannot be clearly displayed by CEUS for sound shadow occlusion (14) (Figure 1).

### 2.2. Clinical outcome

Patients were divided into two groups based on the presence or absence of symptoms: asymptomatic group and symptomatic group. Patients with a history of IS within the previous 2 months belonged to the symptomatic group and had confirmed ischemic lesions in the ipsilateral side's carotid region (anterior circulation) by head CT/MRI. (15). On the other hand, the asymptomatic group consisted of patients without any prior history of stroke to the carotid artery.

### 2.3. Ultrasound procedure

All B-mode US and CEUS examinations were performed by vascular sonographers with at least 5 years of experience in B-mode US and at least 2 years of experience in CEUS of carotid plaques. The CEUS examinations were performed on Philips EPIQ Elite (Philips Healthcare, Amsterdam, the Netherlands) with a high-frequency probe (eL18-4, MHz). We adjusted the frame rate to 12 frames per second and the mechanical index to 0.13 in order to minimize the destruction of microbubbles. The focus location was fixed at the carotid artery's level, and the image depth was adjusted to 3–5 cm based on the carotid artery's size. The B-mode US also used LOGIQ-E9 (GE, Fairfield, USA), Aplio 500 (Toshiba, Japan), and Aplio i900 (Canon, Japan). The vascular sonographers were blinded to the neurologist's findings. All study participants were examined by B-mode US, and the C-IMT was measured. Thereafter, the maximum longitudinal section was considered for CEUS imaging. CEUS was performed after injection of a 1.0-ml bolus of SonoVue solution (Bracco, Milan, Italy) mixed with 5 ml of saline through a peripheral vein. A timer was started to continuously observe the enhancement of CP in real time and store the dynamic examination image. All videos (at least 2-min duration) and images were digitally stored on magnetic optical discs for offline analysis.

### 2.4. Ultrasound image evaluation

**Figure 2:**
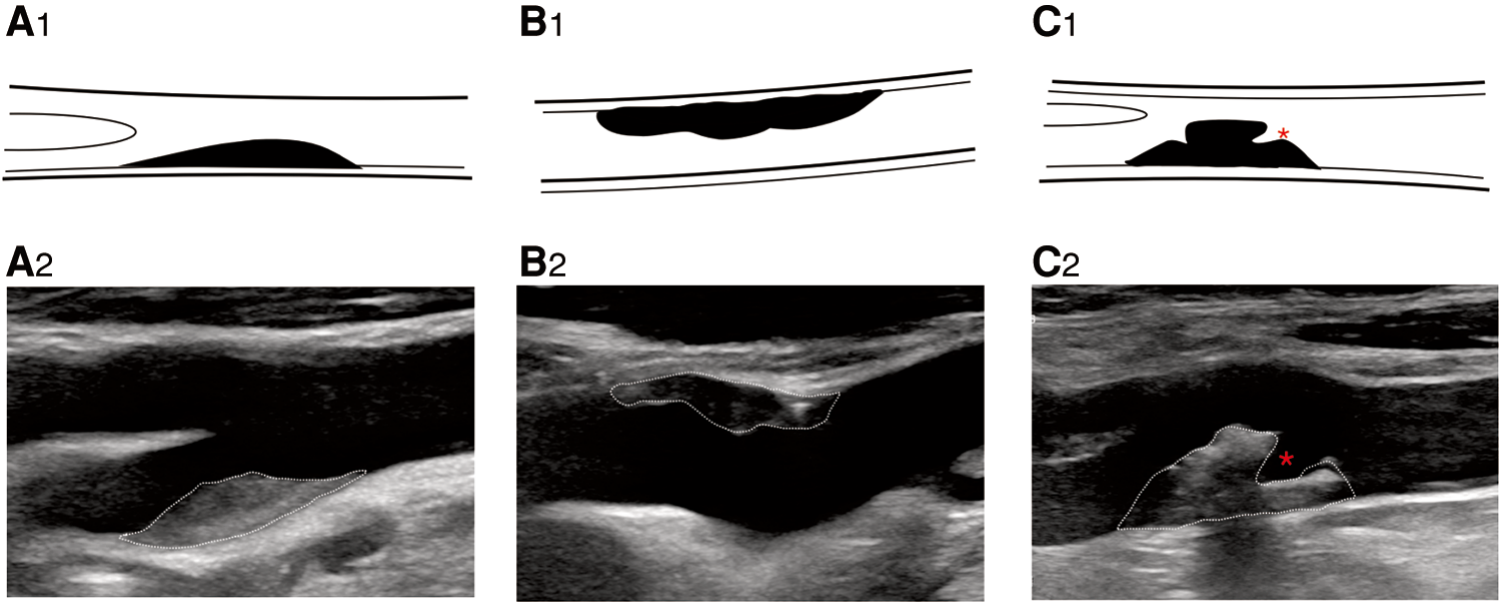
B-mode ultrasound image shows plaque morphology. B-mode ultrasound image shows plaque morphology. (**A_2_**) Regular plaque, (**B_2_**) irregular plaque, and (**C_2_**) ulcerative plaque. * represents plaque ulcer location. (**A_1_–C_1_**) Corresponding schematic diagram.

**Figure 3:**
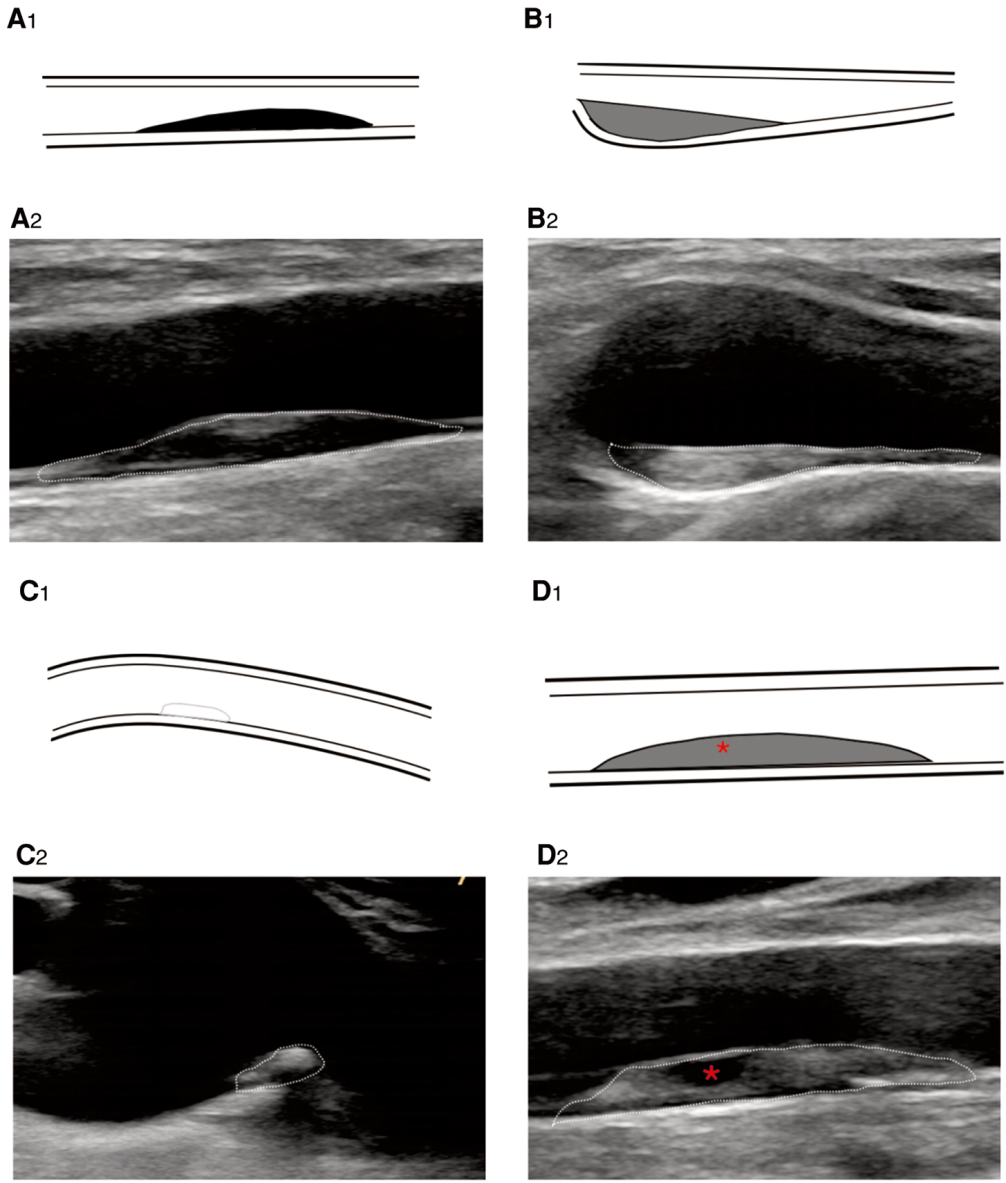
B-mode ultrasound image shows plaque echogenicity. (**A_2_**) Hypoechoic, (**B_2_**) isoechoic, (**C_2_**) hyperechoic, and (**D_2_**) very hypoechoic. * represents very hypoechoic area. (**A_1_–D_1_**) Corresponding schematic diagram.

**Figure 4:**
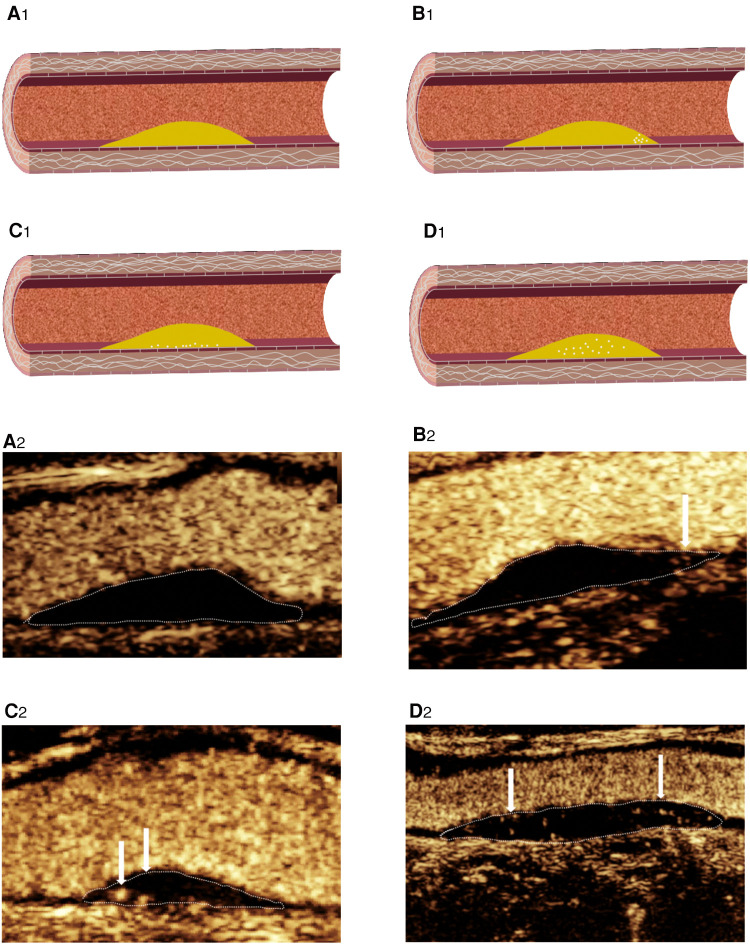
CEUS shows IPN grading. (**A_2_**) Grade 0: no visible microbubbles in plaques. (**B_2_**) Grade 1: minimal microbubbles confined to the shoulder. (**C_2_**) Grade 1: minimal microbubbles confined to the adventitial side of the plaque. (**D_2_**) Grade 2: microbubbles present throughout the plaque core. (**A_1_–D_1_**) Corresponding schematic diagram.

**Figure 5:**
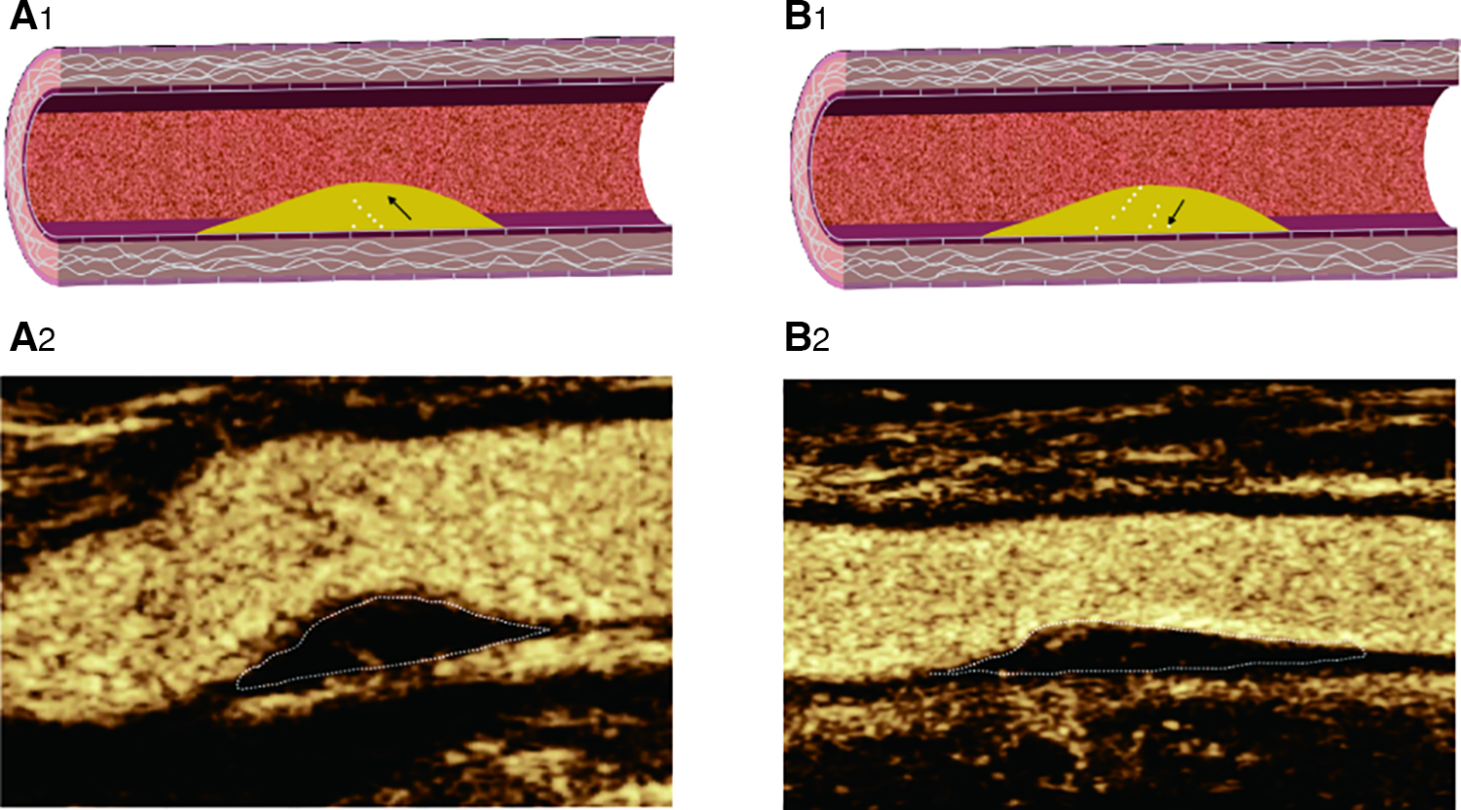
CEUS shows IPN perfusion pattern: (**A**_2_) basal perfusion: microbubbles entered the plaque from the base; (**B**_2_): surface perfusion: microbubbles entered the plaque from the surface; (**A**_1_–**B**_1)_: Corresponding schematic diagram.

The selection of carotid artery plaques was based on the Mannheim consensus (16). According to the perspectives and guidelines of the ASNR Vessel Wall Imaging Study Group and Expert Consensus Recommendations of the American Society of Neuroradiology (17), we abstracted the US features, plaque morphology (irregular, regular) (Figure 2), and echogenicity (hyperechoic, isoechoic, hypoechoic, extremely hypoechoic) (Figure 3). IPN was identified by rapid movement of the echogenic reflectors of microbubbles within the plaque as follows: Grade 0 (no visible microbubbles within the plaque), Grade 1 (minimal microbubbles confined to the shoulder or adventitial side of the plaque), and Grade 2 (microbubbles present throughout the plaque core) (Figure 4). Perfusion pattern was divided into basal entry and surface entry (18) (Figure 5).

Interobserver consistency in IPN grading was analyzed by two independent radiologists (FL and L-NZ) who were blinded to each other's interpretations. To evaluate intra-observer consistency, the data were reanalyzed by the same radiologist (FL) after an interval of 1 month without reference to the initial results.

### 2.5. Essen stroke risk score

The specific rules of the ESRS include eight scoring risk factors, with a maximum score of 9 points (19) (Table 1).

**Table 1:** Essen stroke risk scores of patients.

Risk factor	0 point	1 point	2 points
Age (years)	<65	65–75	>75
Hypertension	N	Y	
Diabetes mellitus	N	Y	
Previous myocardial infarction	N	Y	
Other heart diseases (excluding myocardial infarction and atrial fibrillation)	N	Y	
Smoking	N	Y	
Peripheral arterial disease	N	Y	
Transient ischemic attack	N	Y	

### 2.6. Statistical analysis

A statistical analysis was performed using SPSS 22.0 and the rms packages in R 4.1.0 (The R Foundation for Statistical Computing, Vienna, Austria). Continuous variable data were represented as mean ± standard deviation, and normality testing was performed by using Student's *t*-test. The chi square test was used to assess the relationship between categorical variables. A binary logistic regression model was used to evaluate the relationship between cerebrovascular events and characteristic plaques and the influence of various patch characteristics on the results. The ESRS and C-IMT were analyzed by using the receiver operating characteristic (ROC) curve, and the best cutoff value was determined by the maximum Youden index (defined as sensitivity + specificity − 1). A *p*-value of <0.05 was considered statistically significant.

The prediction nomogram based on the multivariate logistic regression analysis results was developed using R software. The ROC analysis was used to evaluate the discrimination performance of the model. Calibration curves were plotted via bootstrapping using 1,000 resamples. The decision curve analysis (DCA) was performed to determine the clinical usefulness of the model by quantifying the net benefit at the different thresholds.

## 3. Results

### 3.1. Patient baseline characteristics

A total of 824 patients with carotid artery plaques were recruited in the primary cohort. Sixteen patients with severe stroke, 35 patients who did not undergo MRA or CTA above the aortic arch, two patients with carotid artery dissection, four patients with Takayasu's arteritis, 18 patients with poor US imaging quality, and 44 patients with incomplete clinical data were excluded. Finally, a total of 705 patients were included in the study, of which 394 patients belonged in the symptomatic group and 311 patients belonged in the asymptomatic group, as presented in Figure 1. The basic characteristics of the included patients are listed in Table 2.

**Table 2:** Patient characteristics in primary cohort.

	Symptomatic (*N* = 394)	Asymptomatic (*N* = 311)	*p--*value
Age, years^a^^,^^b^	71.03 ± 10.48	65.13 ± 10.31	<0.001
BMI^a^	23.8 ± 4.46	23.68 ± 4.21	0.73
Sex (male), *n* (%)^b^^,^^c^	320 (81)	198 (64)	<0.001
Hypertension, *n* (%)^b^^,^^c^	337 (86)	226 (73)	<0.001
Diabetes mellitus, *n* (%)^c^	166 (42)	124 (40)	0.19
Hyperlipidemia, *n* (%)^b^^,^^c^	158 (40)	87 (28)	0.01
Smoking history, *n* (%)^c^	166 (42)	102 (33)	0.16
Coronary artery disease, *n* (%)^b^^,^^c^	142 (36)	18 (6)	<0.001
Drinking history, *n* (%)^c^	115 (29)	71 (23)	0.09
ESRS^a^^,^^b^	4.46 ± 0.99	2.8 ± 1.09	<0.001
TG, mmol/L^a^^,^^b^	1.71 ± 0.68	1.30 ± 2.11	0.01
TC, mmol/L^a^	4.25 ± 1.11	4.13 ± 1.22	0.21
LDL-C, mmol/L^a^^,^^b^	2.95 ± 0.82	2.72 ± 0.91	0.01
HDL-C, mmol/L^a^	1.20 ± 0.81	1.24 ± 9.15	0.26
Homocysteine^a^^,^^b^	14.11 ± 13.91	12.39 ± 7.06	0.04
hs-CRP, mg/L^a^	9.04 ± 13.91	7.56 ± 7.06	0.54
Creatinine, µmol/L^a^	123.42 ± 26.66	105.89 ± 17.92	0.12
Uric acid, µmol/L^a^	346.7 ± 131.15	342.6 ± 165.93	0.64

BMI, body mass index; TG, triglyceride; TC, total cholesterol; LDL-C, low-density lipoprotein cholesterol; HDL-C, high-density lipoprotein cholesterol; hs-CRP, high-sensitivity C-reactive protein.

^a^ Data are represented as means ± SD.

^b^ *p* < 0.05 indicates statistical significance.

^c^ Data indicate the number of patients, with percentages in parentheses.

### 3.2. Cutoff value for C-IMT

The mean C-IMT was the mean thickness of bilateral common carotid arteries, carotid bifurcations, and the beginning of internal carotid arteries. The mean C-IMT of symptomatic and asymptomatic groups was 2.89 ± 1.14 mm and 2.55 ± 1.06 mm, respectively. The difference between the groups was statistically significant (*p *< 0.001). The optimal coefficient of variation (COV) of C-IMT was 2.25 and 4.55 mm, and the ROC curve analysis showed that the AUC of C-IMT was 0.621 (95% CI: 0.578–0.663).

### 3.3. Cutoff value for ESRS

The ESRS of the symptomatic group and the asymptomatic group were 4.46 ± 0.99 and 2.81 ± 1.09, respectively, with a statistically significant difference of *p* < 0.001. The best COV of the ESRS was 3.5, and the ROC curve analysis showed that the AUC of the ESRS was 0.634 (95% CI: 0.579–0.662).

### 3.4. Establishment ischemic stroke model and development of the nomogram

**Figure 6:**
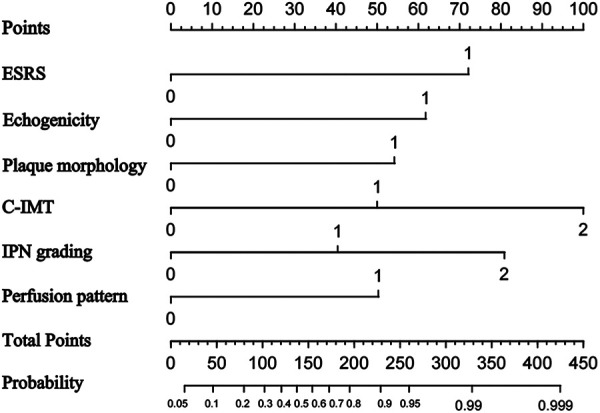
Nomogram to predict ischemic stroke. The nomogram incorporated ESRS, plaque echogenicity, plaque morphology, C-ITM, IPN grading, and perfusion pattern.

We used univariable logistic regression analysis to determine the US features for multivariable logistic regression analysis at a significance level of 0.05. By rounding the mean of regression coefficients of the multivariable logistic analysis to obtain scores for CP risk stratification, we established the CP score according to the parameters from B-mode US and CEUS. Thereafter, we combined the CP score with the ESRS to establish a IS risk model. These variables were used to construct the predictive nomogram (Figure 6).

### 3.5. Establishment of ischemic stroke risk stratification

Based on the mean of regression coefficients of the multivariable logistic analysis of the data in Table 2, the B-mode US features such as hypoechoic, extremely hypoechoic, and irregular shape were assigned 1 point, a C-IMT between 2.23 and 4.55 mm was assigned 1 point, and a C-IMT of greater than 4.55 mm was assigned 2 points. In CEUS, Grade 1 was assigned 1 point, Grade 2 was assigned 2 points, and the perfusion pattern from surface perfusion was assigned 1 point. The ESRS between 3.5 and 8 was assigned 2 points (Table 3).

**Table 3:** Results of univariable and multivariable analysis for various B-mode ultrasound and CEUS features.

	Symptomatic (*N* = 394)	Asymptomatic (*N* = 311)	Univariable analysis		Multivariable analysis		Score^c^
			*β*	*p-*Value^a^	*β* ^b^	*p-*Value^a^	
Echogenicity							
Hyperechoic/isoechoic	140	171	NA				
Hypoechoic/very hypoechoic	277	117	1.06	<0.001	1.43	<0.001	1
Plaque morphology							
Regular	222	252	NA				
Irregular	172	59	1.2	<0.001	1.34	0.07	1
Plaque location							
Common carotid artery	143	131					
Internal carotid artery	100	73	0.23	0.25			
Bifurcation of common carotid artery	151	107	0.26	0.14			
Calcification							
No calcification	304	224					
Calcification	90	87	−0.27	0.12			
C-IMT							
1.5–2.25	123	175					
2.25–4.55	175	95	0.97	<0.001	1.42	0.001	1
>4.55	96	41	1.21	<0.001	2.2	0.001	2
IPN grading							
0	93	125		0.00			
1	76	44	0.84	<0.001	1.39	0.01	1
2	104	29	1.57	<0.001	2.07	0.02	2
Perfusion pattern							
Basal perfusion	79	64					
Surface perfusion	101	9	2.207	<0.001	1.32	0.01	1
ESRS							
0–3.5	120	169					
3.5–8	274	142	1.00	<0.001	1.79	<0.001	2

Unless otherwise specified, data represent the number of nodules.

NA, not applicable.

^a^ Determined by logistic regression analysis.

^b^ Data are means, with values in parentheses indicating 95% CIs of regression coefficients of significant predictive features after 10-fold cross-validation.

^c^ Scoring criteria for significant independent predictors were based on the severely rounded mean of regression coefficients after 10-fold cross-validation to the nearest integer.

**Figure 7:**
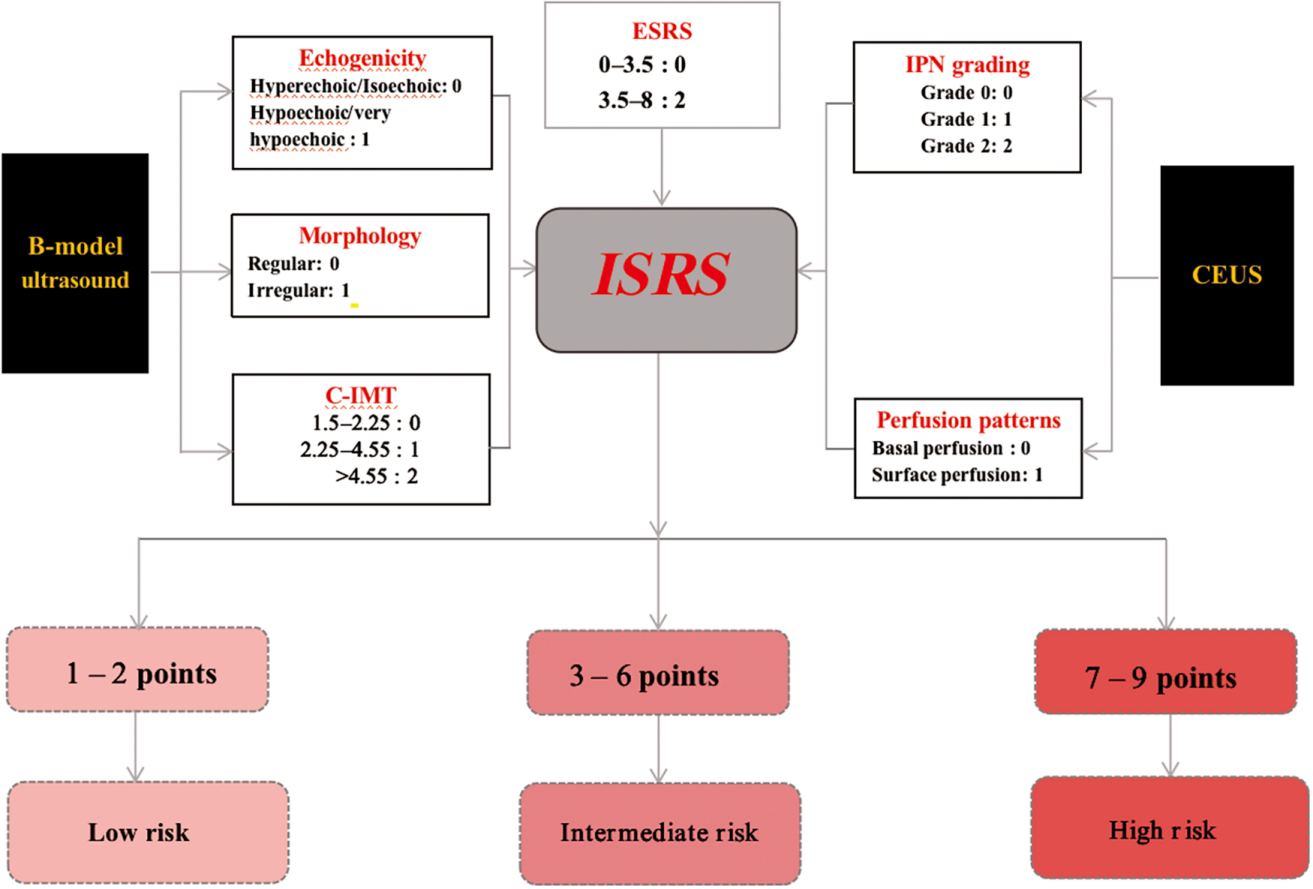
Establishment of the ISRS.

The sum of the points determined the ischemic stroke risk stratification (ISRS): 0–2 points corresponded to CERS 1 (low risk), 3–6 points indicated CERS 2 (intermediate risk), and 7–9 points indicated CERS 3 (high risk) (Figure 7).

### 3.6. Performance of ischemic stroke risk model

**Figure 8:**
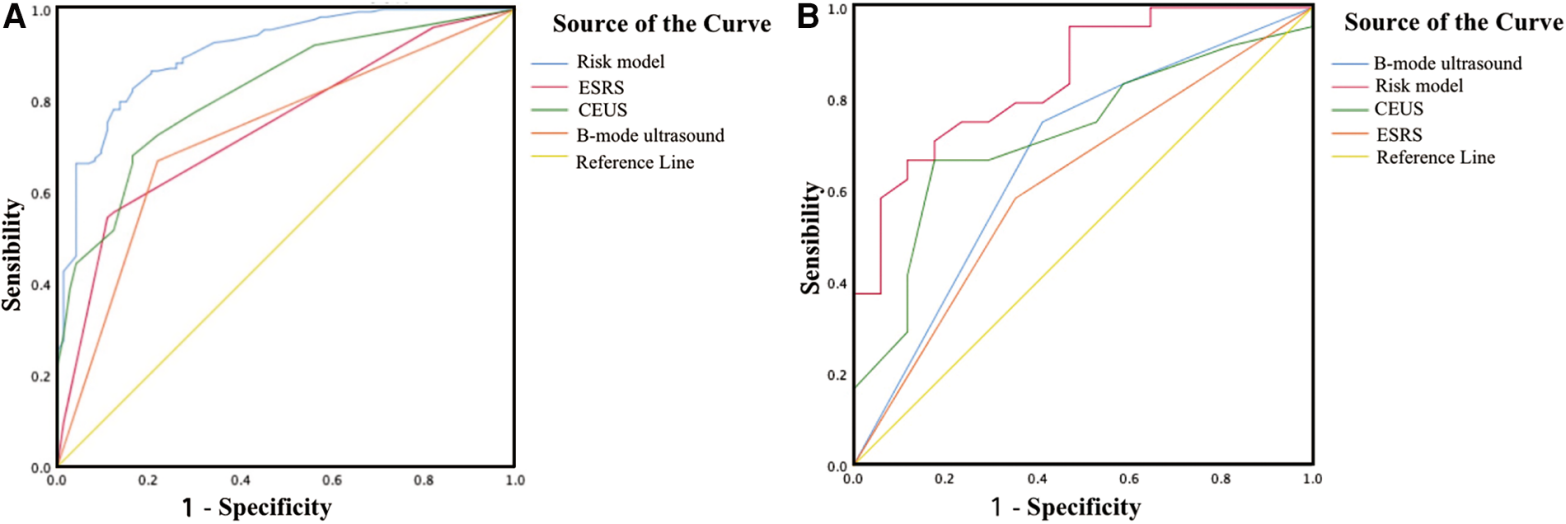
Graph shows the ROC curve. (**A**) ROC curve of the ischemic stroke risk model in the primary cohort. (**B**) ROC curve of the ischemic stroke risk model in the validation cohort.

**Figure 9:**
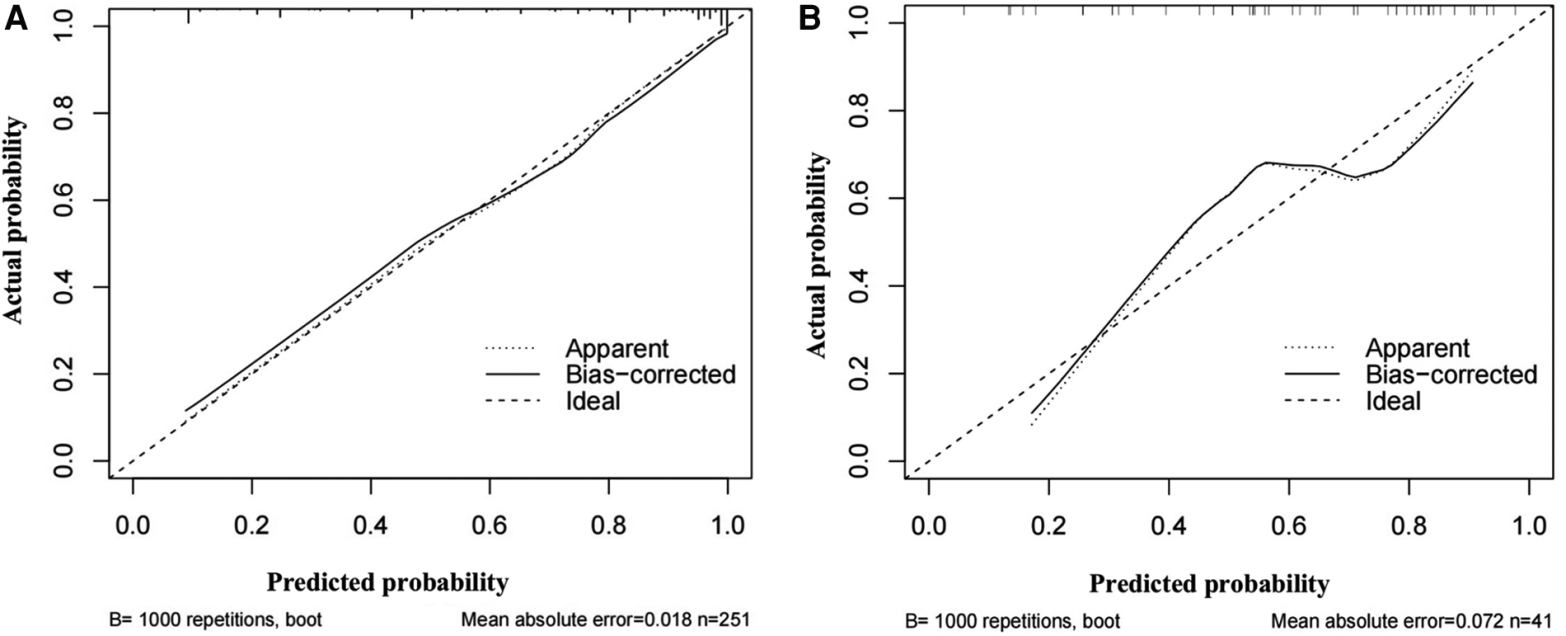
Graph shows the calibration curves of the risk nomogram. (**A**) Calibration curve of the ischemic stroke risk model in the primary cohort. (**B**) Calibration curve of the ischemic stroke risk model in the validation cohort.

The areas under the ROC curve of the model, CEUS, B-mode US, and ESRS in IS were 0.91, 0.82, 0.74, and 0.73, respectively, with statistically significant difference of *p *< 0.001 (Figure 8A). The sensitivity and specificity of the model were 72.6% and 89.3%, respectively. The Hosmer–Lemeshow test illustrated good calibration (*p *= 0.903). The calibration curve of the ischemic stroke risk model demonstrated good agreement between prediction and observation in the primary cohort (Figure 9A).

### 3.7. Validation of ischemic stroke risk model

Internal validation: Using the 68 patients diagnosed with carotid atherosclerosis between January 2023 and April 2023 as the internal validation dataset. The areas under the ROC curve of the model, CEUS, B-mode US, and ESRS in IS were 0.84, 0.72, 0.67, and 0.62, respectively, with statistically significant difference of *p *< 0.001 (Figure 8B). The Hosmer–Lemeshow test yielded a non-significant statistics of *p* = 0.886. Good calibration via bootstrapping using 1,000 resamples was observed for the probability of the ischemic stroke risk in the validation cohort (Figure 9B).

### 3.8. Clinical use

**Figure 10:**
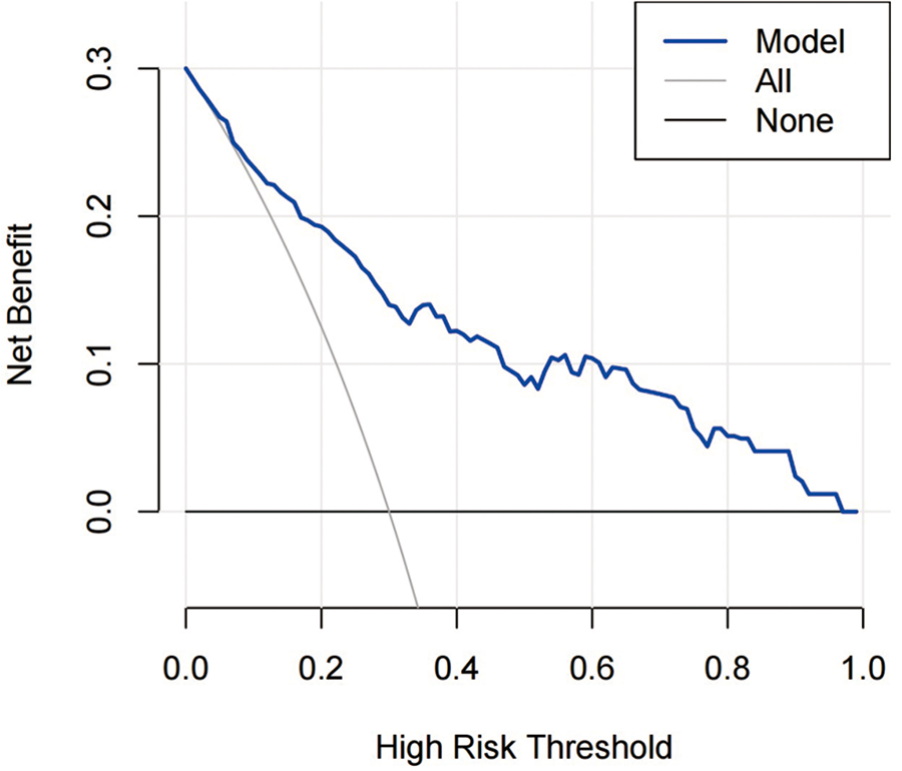
DCA for the risk nomogram. DCA shows that the model is clinically useful when intervention is decided in the threshold range of 8%–100%.

The decision curve analysis for the IS risk model is presented in Figure 10. The decision curve showed that if the threshold probability of a patient or doctor is 8%, using the model to predict ischemic stroke adds more benefit than either the treat-all-patients scheme or the treat-none scheme.

### 3.9. Inter-reader agreement of B-mode US and CEUS features

The IPN grading and perfusion patterns in CEUS showed substantial inter-reader agreement (*k* coefficients = 0.744 and 0.721, respectively), and echogenicity, plaque morphology, plaque location, calcification, and C-IMT in B-mode US showed almost-perfect inter-reader agreement (*k* coefficients = 0.911, 0.901, 0.891, 0.903, and 0.881, respectively).

## 4. Discussion

We combined the B-mode US and CEUS to analyze the characteristics of vulnerable carotid plaques and established the CP score. We developed and validated a IS risk model combining this score and ESRS and established the ISRS to facilitate a clinical work. The area under the curve was 0.907, which indicated practicability and effectiveness of the study.

The ESRS, which includes age, hypertension, diabetes mellitus, smoking status, and peripheral cardiocerebral artery disease, is the most straightforward and extensively used scale in clinical practice to predict the recurrence of IS (20). We observed that the ESRS is an independent risk factor for IS. Weimar et al. (21) found that patients with an ESRS of ≥3 had a significantly higher risk of stroke recurrence and cardiovascular death. We also found that the best COV of the ESRS was 3.5, which prompt for higher IS risk.

US is a non-invasive, easy-to-use, and inexpensive imaging modality. It is critical for assessing C-IMT, echogenicity, and morphology. Our research showed that the optimal COV of C-IMT was 2.25 and 4.55 mm, and the ROC curve analysis showed that the AUC of C-IMT was 0.621. Increased C-IMT has been exhibited to be a risk factor for stroke (22), which is consistent with our research.

We observed that the majority of the plaques in the symptomatic group were hypoechoic or extremely hypoechoic. This is in line with earlier research on the connection between plaque echogenicity and the risk of ischemic events (23), which states that the majority of hypoechoic plaques are lipid-rich plaques that are vulnerable to bleed and rupture when exposed to blood flow (24). Plaque morphology is also one of the risk factors for cerebrovascular events. The shape of the plaques determines the distribution of hemodynamic force, which can be used as the trigger force for plaque rupture and affects the nature and composition of the plaque itself (25). Moreover, some irregular plaques are ulcerative, which can be easily complicated with superficial thrombus to block blood vessels (26).

The plaques of patients in the symptomatic group showed that most of them were above the second grade of IPN in CEUS. The multivariate regression analysis showed that a higher grade of IPN was associated with a higher score in the multivariate regression analysis. IPN secondary to inflammation or hypoxia may be the “precursor” of plaque vulnerability and bleeding within the plaque (27). Song et al. (28) investigated the association between IPN assessed by CEUS and the risk of stroke and showed that IPN is a risk factor for the clinical symptoms of stroke. For these, we used the scores based on the correlation coefficients of different IPNs to reflect plaque vulnerability risk.

The multivariate regression analysis showed that surface perfusion was associated with a higher score in the multivariate regression analysis. It is also an independent risk factor for IS. The pattern of plaque surface perfusion is related to the formation of new blood vessels and the stability of the plaque in CEUS (29). A hydrodynamic analysis sees that the diffusion of contrast agents from the surface perfusion can be considered as the rupture of the fiber cap can cause blood to enter the plaque (30). Moreover, local damage to the skin cells on the inner membrane surface can induce local inflammation and promote the growth of new blood vessels from the surface perfusion (31).

This is the first risk model that incorporates US imaging and ESRS for the ISRS in patients with low and intermediate carotid stenosis. Our study contains some strengths. First, our approach makes scoring simple and convenient without the need for complicated calculations or conversion processes. Second, due to the inclusion of patients who underwent B-mode US as well as those who underwent CEUS, our dataset balances out the selection bias that could be caused by including the data of only CEUS. Third, quantitative CEUS features and B-mode US traits are included in the assessment criteria to indicate CP vulnerability, and the grading criteria for each feature are based on their rounded regression coefficients.

However, our study also has some limitations that should be considered. First, we used a sample from a single center, which is less applicable to an entire population than a multicenter sample would be. Second, the majority of the study data are from the cardiovascular and cerebral clinic which makes selection bias inevitable. Finally, a number of influencing factors for IS could not be extensively explored. The prevalence of the aforementioned concerns could skew the outcomes of this study and make them less objective and accurate. The model has to be prospectively validated at numerous centers before being deployed in clinical settings.

## 5. Conclusion

The ISRS based on B-mode US, CEUS features, and ESRS in patients with low and intermediate carotid stenosis is of great significance for hierarchical management, stroke prediction, and risk warning in patients with atherosclerosis.

## Data Availability

The raw data supporting the conclusions of this article will be made available by the authors, without undue reservation.
